# Case Report: Gefitinib in EGFR 19del recurrent aggressive fibromatosis

**DOI:** 10.3389/fonc.2025.1537714

**Published:** 2025-07-14

**Authors:** Yanjing Guo, Jingjing Wu, Qianming Bai, Juefeng Wan, Qifeng Wang, Xinhong He, Xiaowei Zhang, Zhiguo Luo, Liju Xing, Xin Liu

**Affiliations:** ^1^ Department of Medical Oncology, Fudan University Shanghai Cancer Center, Shanghai, China; ^2^ Department of Oncology, Shanghai Medical College, Fudan University, Shanghai, China; ^3^ Department of Multidisciplinary Oncology, Fuzhou First General Hospital Affiliated with Fujian Medical University, Fujian, China; ^4^ Department of Pathology, Fudan University Shanghai Cancer Center, Shanghai, China; ^5^ Department of Radiation Oncology, Fudan University Shanghai Cancer Center, Shanghai, China; ^6^ Shanghai Clinical Research Center for Radiation Oncology, Shanghai Key Laboratory of Radiation Oncology, Shanghai, China; ^7^ Department of Interventional Radiology, Fudan University Shanghai Cancer Center, Shanghai, China; ^8^ Department of Oncology, Jiangyin Traditional Chinese Medicine Hospital, Jiangyin, China

**Keywords:** aggressive fibromatosis, EGFR 19del, targeted therapy, gefitinib, partial response

## Abstract

We present the first case of a male patient with an epidermal growth factor receptor (EGFR) 19del mutation who was diagnosed with intra-abdominal aggressive fibromatosis and familial adenomatous polyposis. We assessed the clinical response of the patient to first-generation EGFR-tyrosine kinase inhibitors (EGFR-TKIs). A remarkable sustained partial response was achieved with the application of gefitinib after progression on multiple lines of therapy. The main adverse event of gefitinib treatment in this patient was a grade 2 rash. (Funded by the National Natural Science Foundation of China [Grant No. 82003061] and the Shanghai Sailing Program [20YF1408800] to Yanjing Guo, the Natural Science Foundation of Shanghai [Grant No. 24ZR1412800] to Xin Liu).

## Introduction

Aggressive fibromatosis (AF) is a rare proliferative tumor of fibroblasts/myofibroblasts originating from soft tissues. It was first described by McFarlances in 1832 and later termed as desmoid tumor or desmoid fibromatosis (1). Depending on the location of tumor growth, AF can be classified into three types: extra-abdominal, abdominal wall, and intra-abdominal (2). It is further categorized into familial adenomatous polyposis (FAP)-related and sporadic types based on its association with FAP. FAP-related AF exhibits familial clustering and is more commonly found in the abdominal cavity (3), while sporadic AF is often associated with CTNNB1 gene mutations (4).

Clinical guidelines recommend a wait-and-see approach for patients who are asymptomatic, have smaller tumors, slow tumor growth, or no organ dysfunction. Currently, the radical treatment for AF is extended surgical resection with a negative margin, despite local recurrence rates as high as 30%–40% (5). Radiotherapy can also be used to treat AF, contributing to long-term local control (6). Systemic therapy, including endocrine drugs, non-steroidal anti-inflammatory drugs (NSAIDs), chemotherapy, and targeted therapeutic agents, such as anti-VEGF-targeted drugs and γ-secretase inhibitors, can improve clinical outcomes in patients with refractory AF unsuitable for surgery or radiotherapy (7, 8). With the application of genetic testing technology, next-generation sequencing (NGS) offers novel insights into genetic variations and potential targeted therapies for AF.

Herein, we reported the first case of a patient with AF carrying an epidermal growth factor receptor (EGFR) exon 19 deletion mutation who was diagnosed with FAP and had unresectable intra-abdominal AF. A notable and sustained partial response was observed in this patient treated with gefitinib after multiple lines of treatment.

## Methods

### Administration of gefitinib and clinical assessments

The patient provided informed consent for targeted therapy and received gefitinib at a dose of 250 mg orally once daily. Adverse events were documented according to the National Cancer Institute’s Common Terminology Criteria for Adverse Events (v5.0), and clinical response was assessed using the Response Evaluation Criteria in Solid Tumors version 1.1 (9, 10).

### Next-generation sequencing and data analysis

Details on NGS and data analysis are provided in the **Supplementary Methods** section of the **Supplementary Appendix**.

### Case report

A 32-year-old Asian man with a family history of FAP was diagnosed with rectal cancer through colonoscopy and biopsy. The patient underwent high anterior proctectomy and terminal ileostomy with excision of a 17 cm bowel on 13 January 2016. Pathological examination revealed a well-to-moderately differentiated adenocarcinoma with tumor infiltration into the submucosa. All incisal margins were negative without microscopic vascular or perineural invasion. During surgery, 16 lymph nodes were dissected, and no metastases were observed. The patient was clinically and pathologically classified as T1N0M0, stage I. Neither chemotherapy nor radiotherapy was administered postoperatively. In May 2016, the patient underwent a stoma retrieval procedure. In May 2019, total colectomy was performed, preserving the anus and rectum approximately 3 cm in length, and ostomy reinnervation was conducted 5 months later. Subsequent colonoscopies were conducted regularly, and polypectomy was performed under endoscopic guidance.

The patient was admitted to our hospital with abdominal pain in January 2022. A pre-admission full-body computed tomography (CT) scan revealed a large tumor mass in the right abdominopelvic cavity, which was indistinguishable from the metastatic and interstitial tumors of the small intestine. The right ureter was obstructed owing to compression by the adjacent tumor mass, and ureteral stents were used by urologists to relieve the obstruction. Routine blood, urine, and stool examinations revealed no obvious abnormalities. Biochemical tests for liver and kidney function, plasma coagulant levels, and tumor marker levels were within normal limits. A post-admission abdominal CT scan on 14 March 2022 revealed a lower abdominal mass measuring approximately 15.0×8.2 cm, along with a nodule measuring about 4.5×3.0 cm in the muscular layer of the left abdominal wall (**Supplementary Figure S1**). Next, CT-guided core needle biopsy of the abdominal mass lesion was performed. The specimens were reviewed by multiple pathologists, all of whom concurred with the diagnosis of AF. Immunohistochemistry and hematoxylin-eosin (HE) staining of the specimens are shown in **Supplementary Figure S2**, with the negative expression of AE1/AE3, CD34, Desmin, DOG-1, SMA, and S-100.

**Figure 1:**
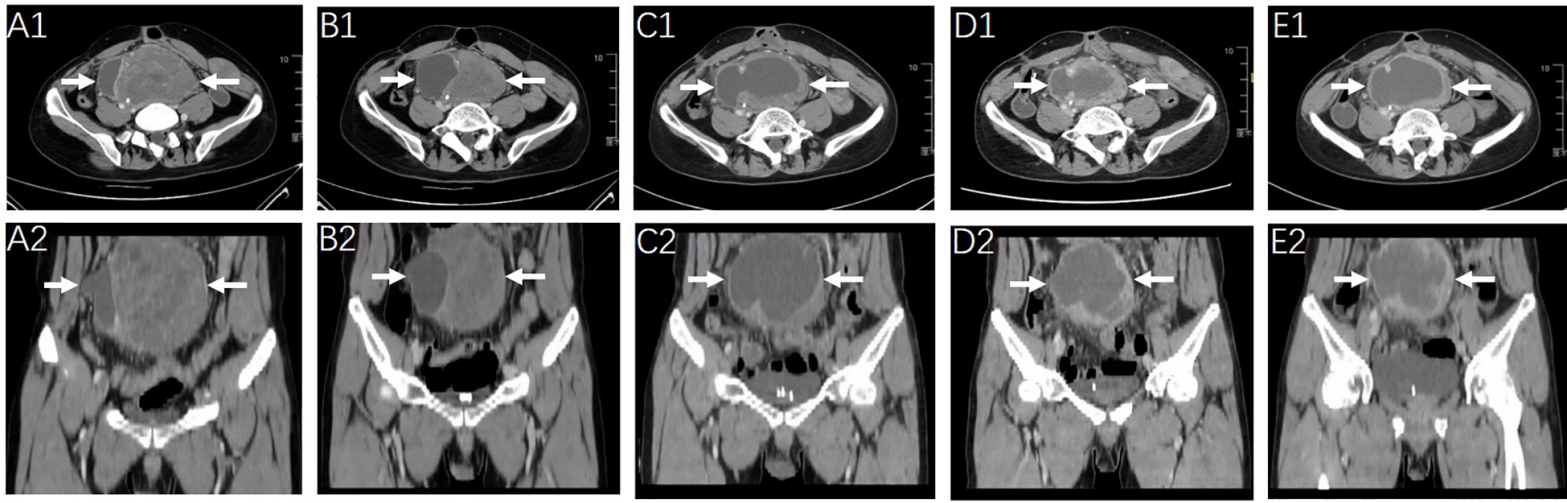
Radiologic response to the first-line treatment of radiotherapy combined with anlotinib and sintilimab monoclonal antibody and the second line treatment of chemotherapy. CT scanning images of the abdominopelvic tumor mass (arrow) were obtained before the initiation of first-line on March 14, 2022 **(A1, A2)** and after 2-months of therapy on May 12, 2022 **(B1, B2)**. Representative images of the abdominopelvic tumor mass (arrow) on July 2022 [before chemotherapy, **(C1, C2)**] September 2022 [after 3 cycles of chemotherapy, **(D1, D2)**] and December 2022 [after 6 cycles of chemotherapy, **(E1, E2)**].

Owing to the large volume of the intra-abdominal lesion and the occurrence of abdominal wall metastasis, surgical treatment was not feasible for this patient, and radiotherapy was administered concurrently with the oral administration of 12 mg anlotinib daily from March 2022 to April 2022. The radiotherapy planning system targeting abdominal lesions is shown in **Supplementary Figure S3**. The patient was then administered 200 mg sintilimab for four cycles until May 2022 after radiotherapy. Imaging studies after 8 weeks of combined treatment indicated notable shrinkage of the tumor masses (**Figures 1, A1–B2**). Two months later, the patient experienced a slight increase in the size of the abdominal mass and more pronounced abdominal distension. Subsequent CT scanning in July 2022 (**Figures 1, C1, C2**) revealed a significant decrease in the solid portion of the mass and an increase in the cystic component, resulting in an overall increase in tumor volume. Therapeutic response was evaluated as progressive disease (PD).

Second-line treatment with cytotoxic agents was initiated, with systemic chemotherapy starting from 7 July 2022 to 17 November 2022. Liposomal doxorubicin (60 mg) and dacarbazine (1000 mg) were administered on the first day and repeated every 3 weeks for a maximum of six cycles. The treatment was completed without severe adverse effects or dose reduction of chemotherapy drugs. Regular CT scanning images after three and six cycles of chemotherapy revealed no significant change in the solid components but a slight increase in the cystic components of the tumor masses and a slight increase in the tumor volume (**Figures 1, C1–E2**). The clinical effect of chemotherapy was evaluated as stable disease (SD).

### Molecular findings

**Figure 2:**
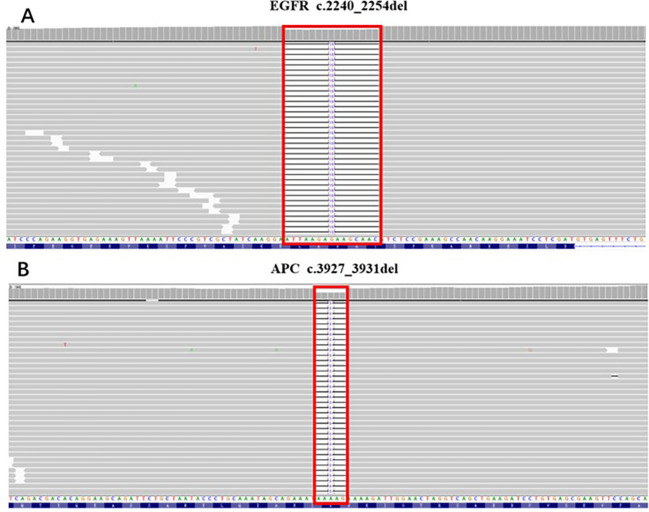
The genomic alterations of APC and EGFR detected in the biopsy specimen. **(A)** EGFR: c.2240_2254del (p.Leu747_Thr751del); AF=12.38%. **(B)** APC: c.3927_3931del (p.Glu1309fs); AF=55.04%.

A comprehensive genomic analysis using a 68-gene universal genomic DNA kit was conducted on biopsy specimens obtained in February 2022. The genomic alterations were shown as follows:

EGFR exon 19 deletion mutation in c.2240-2254del (p.Leu474-Thr751del), AF 12.38% (**Figure 2A**);
APC exon 16 frameshift mutation in c.3927-3931del (p.Glu1309fs), AF 55.04% (**Figure 2B**);
APC exon 15 frameshift mutation in c.5782del (p.Gln1928fs), AF 15.75%;
CDKN2A exon 2 missense mutation in c.344T>A(p.Val115Glu), AF 30.85%.

**Figure 3:**
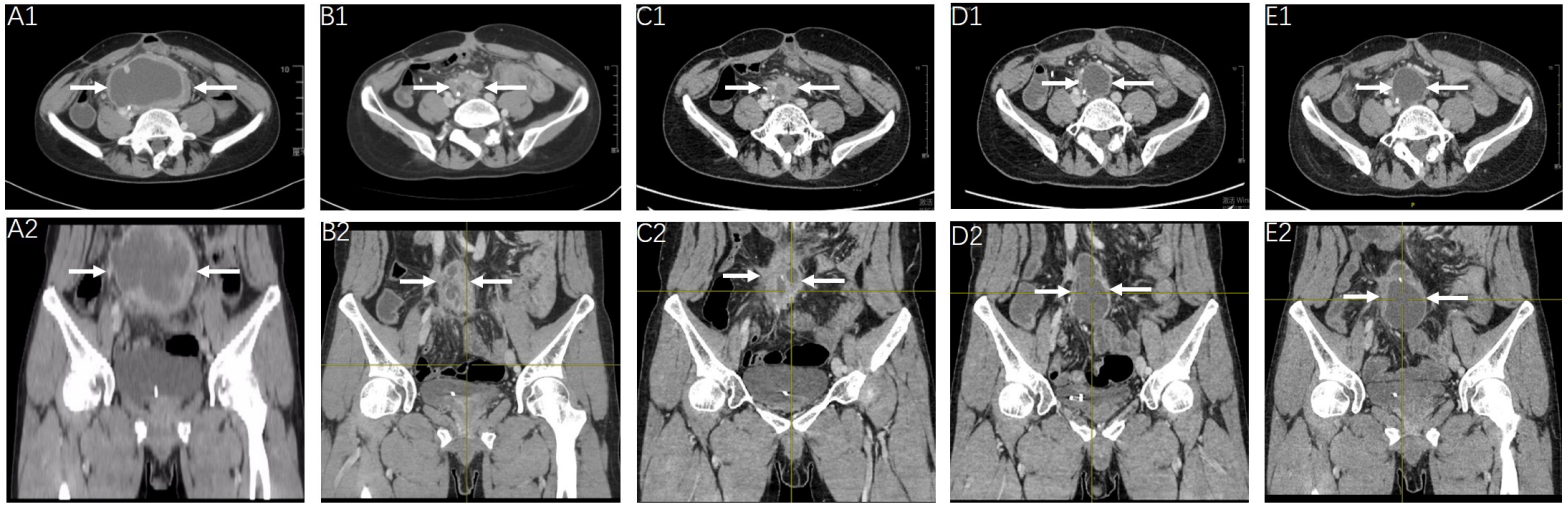
Radiologic response to gefitinib. Clinical efficacy evaluation of gefitinib at the timepoint of February 2023 **(A1, A2)**, July 2023 **(B1, B2)**, October 2023 **(C1, C2)**, March 2024 **(D1, D2)** and May 2024 **(E1, E2)**, showed continued shrinkage of the abdominopelvic tumor mass (arrow).

As recommended by the multidisciplinary team, the following therapeutic strategy should be developed according to the aforementioned NGS results, which showed an EGFR exon 19 deletion mutation. Owing to the failure of previous systemic treatments, accompanied by severe clinical symptoms, the patient accepted the prescribed first-generation EGFR-tyrosine kinase inhibitor (TKI) gefitinib (Iressa 250 mg po. Qd) on 14 February 2023. The tumor mass is shown in **Figures 3, A1, A2**. Marked regression of the tumor mass was observed 3 months after gefitinib administration. Notably, there was over 60% reduction in maximum diameter of the tumor mass after 5, 8, and 13 months of EGFR-TKI treatment, which was evaluated in July 2023, October 2023, and March 2024, respectively (**Figures 3, B1, D2**). The latest clinical efficacy was confirmed in May 2024 with an abdominopelvic tumor mass measured as 4.3×3.7 cm (**Figures 3, E1, E2**). In the safety evaluation, the main toxicity of oral EGFR-TKI medication in patients was grade 2 rash without significant hematologic toxicity.

## Discussion

AF is considered as an intermediate tumor with high heterogeneity and a tendency to invade surrounding tissues, which can lead to local recurrence. The frequent recurrence of AF and multiple lines of therapy can significantly compromise organ function, diminish a patient’s quality of life, and even result in life-threatening complications. Therefore, a proactive therapeutic treatment is recommended for patients with symptomatic or progressive AF. Given the indolent nature of AF, several guidelines recommend a watchful waiting strategy as a preferred option (11). Radiotherapy has also proven to be effective in preventing local recurrence of AF (12). In terms of pharmacological treatments, endocrine therapies, NSAIDs, traditional cytotoxic agents, and targeted therapies have been recommended by multiple guidelines (7, 13). Endocrine therapy and NSAIDs have shown limited efficacy compared to cytotoxic agents, and the adverse effects associated with the latter have affected its routine recommendation (14). Targeted therapies have revolutionized anticancer treatments by improving personalized therapy. Small-molecule TKIs, such as imatinib and sorafenib, have been utilized primarily as salvage treatment and have demonstrated certain clinical efficacy in AF, with overall response rates ranging from 10% to 23% (15, 16). Targeted therapies focusing on the Notch signaling pathway, specifically γ-secretase inhibitors, have shown promise as effective treatments for AF (17, 18). However, nirogacestat is unavailable in China.

However, the pathogenesis of AF remains unclear. FAP-related AF is linked to APC gene deletion and impaired phosphorylation of β-catenin, resulting in aberrant tumor cell proliferation (19). Generally, CTNNB1 gene mutation are detected in 90-95% of sporadic AF cases, leading to the activation of the Wnt/β-catenin signaling pathway and excessive accumulation of β-catenin (4, 20). Mutations in AKT1 and BRAF have also been reported in pediatric AF (21). Novel pathogenic gene mutations have been identified through whole-genome sequencing in patients with AF. The reported gene variations include AKT1 (G311S/D and T312I), ALK (R806H and G924S), AR (A159T), EGFR (P848L), ERBB2 (H174Y), KIT (V559D), RET (T1038A), SDHA (R325M), and SDHD (R115W) (22). Although the literature has reported certain EGFR mutations, no studies have linked EGFR exon 19 deletion mutations to pathogenic variations in AF.

With the continuous advancement of molecular detection technologies in recent years, comprehensive assessments of patients guided by multidisciplinary teams and NGS have facilitated discussions and the development of personalized clinical strategies in the era of precision medicine, benefiting the clinical efficacy for patients with AF (23). A 32-year-old male patient with AF exhibited a rare EGFR exon 19 deletion mutation (AF=12.38%) and an APC mutation (AF=55.04%). He had a history of FAP and had undergone radical surgery and total colectomy for rectal cancer. Years later, the patient presented with abdominal distension and was pathologically diagnosed with AF. Imaging revealed multiple unresectable lesions that were classified as intra-abdominal or FAP-related. In the first-line treatment, radiotherapy combined with anti-angiogenic TKI and immunotherapy was administered with a progression-free survival of about 2 months. In the second-line treatment, six cycles of cytotoxic chemotherapy were employed, and routine imaging evaluation indicated SD for >5 months with slight tumor enlargement. Through multidisciplinary team discussions and NGS of biopsy specimens, an EGFR exon 19 deletion mutation was identified. Multiple experts suggested the use of the EGFR-TKI gefitinib for targeted therapy, and a notable and long-lasting partial response of more than 1 year was observed thereafter. Through a literature review, we report the EGFR exon 19 deletion mutation in AF for the first time, noting that no standard treatment regimen is recommended for this subtype. Drawing on experiences from treating EGFR gene mutations, the most common oncogenic drivers in non-small cell lung cancer (24), EGFR-TKI targeted therapy may be applicable to patients with AF carrying EGFR mutations. Tumor-agnostic therapies represent clinical strategies based on specific genetic anomalies (25). In this case, NGS facilitates the identification of potential gene mutations and promotes the application of potential effective targeted drug of gefitinib in this AF rare tumor. We conclude that patients with rare tumor carrying infrequent gene alterations should have more access to targeted therapies, emphasizing the benefits of tumor-agnostic therapy.

## Conclusion

The management of patients with AF requires a more individualized and flexible approach. The watch-and-wait strategy is preferred for newly diagnosed and asymptomatic patients with AF. However, for patients with rapid disease progression or onset of clinical symptoms, personalized combination therapy should be formulated under the guidance of NGS technologies and multidisciplinary consultations. In summary, the management of AF includes maximizing tumor control, delaying disease progression or recurrence, minimizing treatment-related toxicity, and improving patient survival.

## Data Availability

The data analyzed in this study was obtained from Department of Medical Oncology, Fudan University Shanghai Cancer Center, the following licenses/restrictions apply hospital ethics. Requests to access these datasets should be directed to Xin Liu, jeanettexin@hotmail.com.
