# Supplementary material for: Case Report: Gefitinib in EGFR 19del recurrent aggressive fibromatosis
Source: Front Oncol. 2025 Jul 14;15:1537714. doi: 10.3389/fonc.2025.1537714 (PMC12301188; doi:10.3389/fonc.2025.1537714)
Supplement: Supplementary file 1 [file DataSheet1.docx]

**Supplementary Figures**


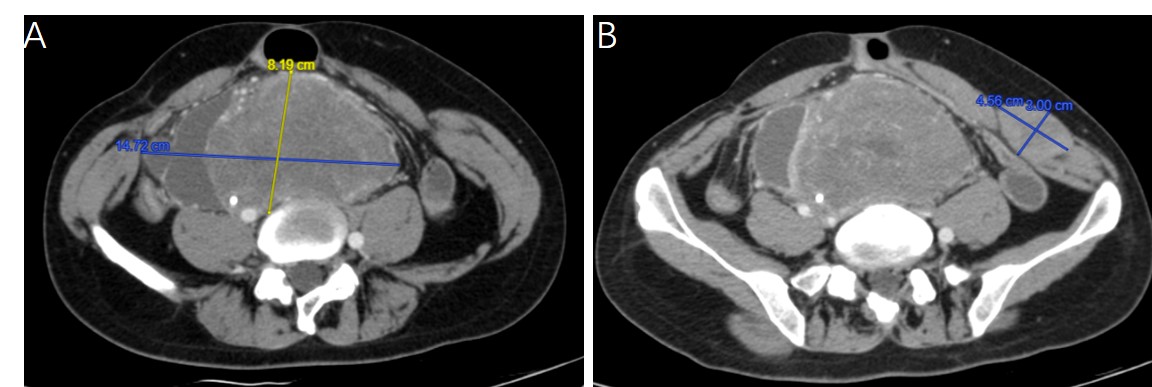


**Supplementary Figure 1**. **Representative images of the tumor mass in the abdominal cavity (A) and on the left abdominal wall (B) on March 14, 2022.**


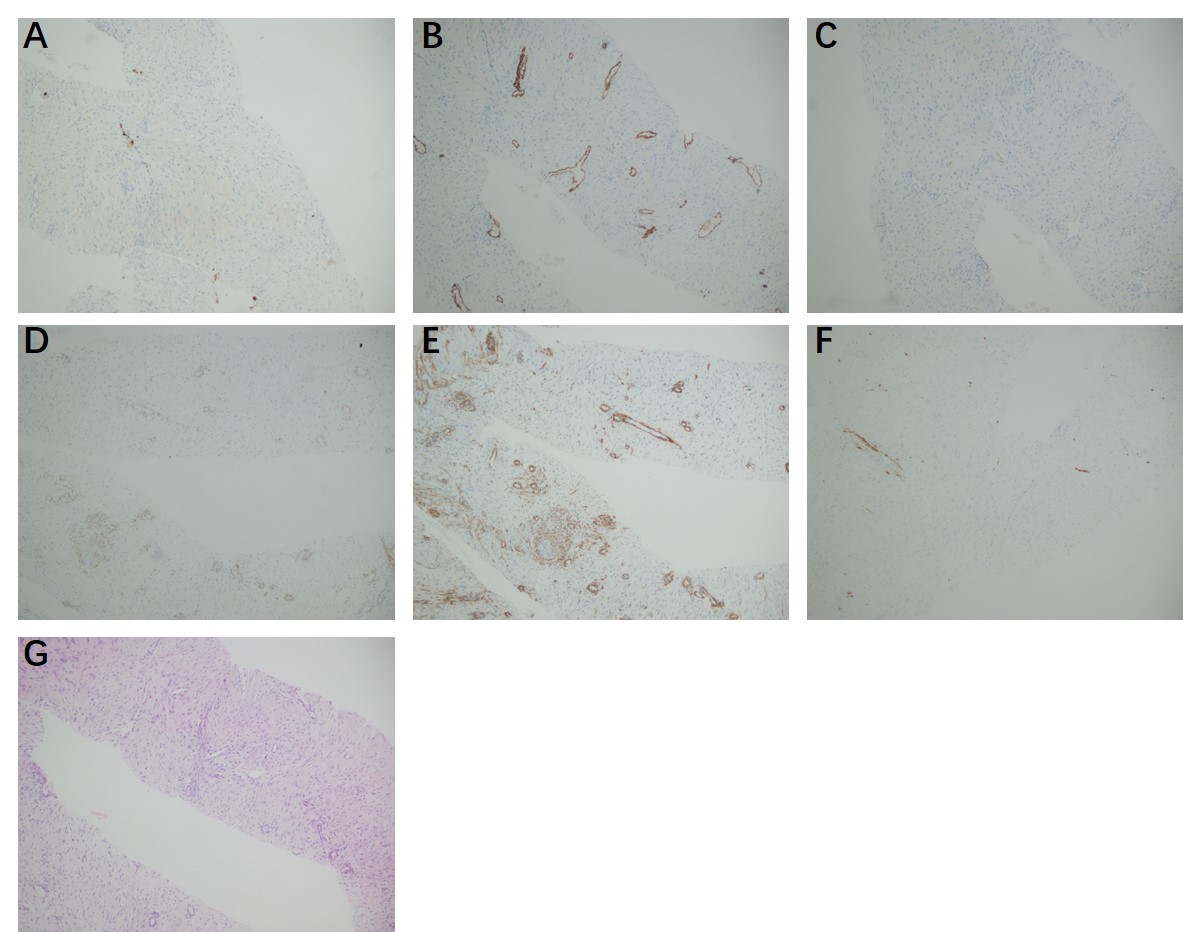


**Supplementary Figure 2. Representative images of immunohistochemistry (IHC) staining (A-F) and hematoxylin-eosin (HE) staining (G).**

**A**. AE1/3, **B.** CD34, **C.** Desmin, **D.** DOG-1, **E.** SMA, **F.** S-100


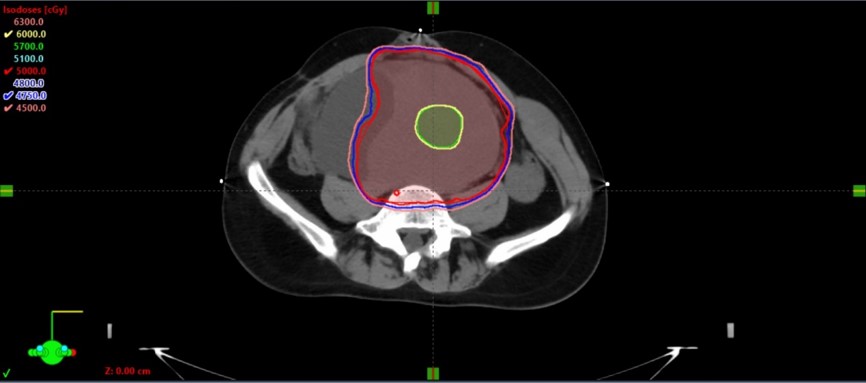


**Supplementary Figure 3.** **Isodose curves used for the integrated boost 7‑gantry angle intensity-modulated radiotherapy planning.**

Radiotherapy Plan: PTV = 50Gy/25Fx, with a simultaneous boost to PTV-boost = 60Gy. The inner line (*red*) represent the 5000c Gy zone，the outer line (*blue*) represents the 4750c Gy zone and the center line(*yellow*) represent the 6000c Gy zone.

**Supplementary Methods of Next-generation sequencing and Data analysis**

Total genomic DNA was extracted from tissue samples using a QIAamp DNA Mini Kit (Qiagen, Hilden, Germany), in accordance with the manufacturer’s instructions. Genomic DNA was profiled using a capture-based targeted sequencing panel (Burning Rock Biotech), which included all exons of 68 genes. The concentration of the DNA samples was quantified using the Qubit dsDNA assay (Thermo Fisher Scientific, Waltham, MA, USA). Subsequently, the DNA was then sheared to 300 bp using a Covaris S220 Focused ultrasonicator (Covaris, Woburn, Massachusetts, USA), This was followed by hybridization with the capture probe baits, hybrid selection with magnetic beads, and PCR amplification. The quality and size range of the DNA were then assessed using the QIAxcel Advanced automated nucleic acid analysis system (Qiagen). Subsequently, the available indexed samples were subjected to sequencing on a NextSeq 550 System (Illumina, San Diego, CA, USA) with paired-end reads. The sequencing data were aligned to the human genome (hg19) using Burrows-Wheeler Aligner version 0.7.10. Local alignment optimization, variant calling, and annotation were performed utilizing the Genome Analysis Toolkit version 3.2 and VarScan version 2.4.3.
